# Comparison of the Full Outline of UnResponsiveness Score and Glasgow Coma Scale in Predicting Endotracheal Intubation, Hospital Length of Stay, and Mortality Among Patients With Non-traumatic Altered Mental Status in the Emergency Department

**DOI:** 10.7759/cureus.94966

**Published:** 2025-10-20

**Authors:** Mohammad Ghaleb Abbas, Ameer Ayad Jawad Al-Musawi, Abdulillah R Khamees, Ghaith M Taha, Rafal Abdulamir Abdullah Almaulla, Omer Faris Nawar, Sama Jamal Baqer, Saja Jaafar Abotaleb, Abdullah Muhanned Isam, Athraa S Ahmed

**Affiliations:** 1 College of Medicine, Nahrain University, Baghdad, IRQ; 2 Department of Medicine, Al-Shaheed Al-Sader Hospital, Baghdad, IRQ; 3 Department of Neurology, Al Kadhimiya Teaching Hospital, Baghdad, IRQ; 4 Department of Medicine, Al Karama Teaching Hospital, Baghdad, IRQ; 5 Department of Medicine, Al Kindy College of Medicine, Baghdad, IRQ; 6 Department of Medicine, Al-Taji Sector of Health Care, Al-Taji First Primary Health Center, Baghdad, IRQ; 7 Department of Medicine, Tarmiyah General Hospital, Baghdad, IRQ; 8 Department of Medicine, Nu'man Teaching Hospital, Baghdad, IRQ

**Keywords:** altered mental status evaluation, full outline of unresponsiveness score, glasgow coma scale score, length of hospital stay (los), endotracheal intubation

## Abstract

Introduction: The Glasgow Coma Scale (GCS) is a commonly used assessment tool; however, it has recognized limitations, particularly in patients who are intubated or have aphasia. The Full Outline of UnResponsiveness (FOUR) score was created to tackle these limitations. It has been studied mainly in the trauma and neuroscience setting; to our knowledge, this is the first study to compare the predictive performance of GCS and FOUR scores for endotracheal intubation and length of hospital stay in non-traumatic altered mental status patients in the emergency department (ED).

Methods: A prospective cohort study was conducted among adult patients from May 2024 to July 2025. The area under the receiver operating characteristic (AUROC) curve was used to evaluate and compare the predictive accuracy of the GCS and FOUR scores in predicting outcomes.

Results: A total of 240 patients were included in the study. The median age was 70 years (IQR: 21); 55.0% were female. In-hospital mortality was 39.6%. 27.9% of patients required ICU admission. The FOUR and GCS scores both demonstrated significant discriminative ability across outcomes (all p < 0.001). For in-hospital mortality, the FOUR score showed superior accuracy (AUROC 0.851, 95% CI: 0.803-0.900) compared to the GCS (0.771, 95% CI: 0.710-0.831). Similarly, in predicting endotracheal intubation, the FOUR score outperformed the GCS (AUROC 0.875 vs. 0.781). For the length of hospital stay, the FOUR score also demonstrated higher predictive value (AUROC 0.771 vs. 0.734).

Conclusion: The FOUR score demonstrated superior predictive accuracy compared to the GCS across all clinical outcomes, including mortality, intubation, and length of hospital stay. It may serve as a more reliable tool for prognostication in non-traumatic patients with altered mental status.

## Introduction

Altered mental status is a broad clinical description including various disturbances in cognitive, behavioral, and consciousness states, such as confusion, disorientation, lethargy, and coma. The emergency department (ED) presentation may be common but complex and is often frank evidence of severe underlying disease or neurological conditions. An early evaluation and outcome prediction of altered mental status are critical for prior course of action intervention and major decisions on the level of care needed for the patient [1,2].

The Glasgow Coma Scale (GCS) is an assessment tool used for evaluation of neurological performance, but it is not comprehensive in categorizing the different states of consciousness [3]. A patient who is intubated cannot speak and hence does not qualify for the verbal component of the scale. Pain withdrawal can be misinterpreted as a flexion response to pain. Eye-opening indicates wakefulness, but not comprehension, as seen in persistent vegetative states. The GCS fails to consider brainstem reflexes, respiration patterns, or the need for mechanical ventilation, all of which could supplement coma severity and thus improve neurological evaluation [3-5].

Wijdicks et al. proposed the Full Outline of UnResponsiveness (FOUR) score as a new coma scale in light of the deficiencies of the GCS [4]. The scale aims to offset the above-listed shortcomings of the GCS. The FOUR score covers this gap since it is more neurologically refined. It is subdivided into four components: eye response, motor response, brainstem reflexes, and respiration. It can define altered states of consciousness not possible by GCS, such as vegetative state and locked-in syndrome, and provides specific information on the respiratory drive and patterns, such as hypoventilation, which may indicate the need to initiate mechanical ventilation in suspected comatose patients. It includes greater neurological detail, including brainstem reflexes and respiratory components, and increases the likelihood of further defining the severity of the lowest three GCS levels. Thus, a more global neurological assessment could improve early decision-making and triage potential [4,6,7].

Ever since the validation of the FOUR score, numerous studies have assessed its performance and compared it with the GCS, particularly in neurocritical care and trauma settings [5,8-10]. However, there remains a significant gap in the literature regarding its utility in evaluating patients with non-traumatic or medically induced altered mental status, especially in ED settings. To the best of the authors' knowledge, this is the first study to compare the utility of the FOUR and GCS scores in endotracheal intubation and the length of hospital stay among non-traumatic altered mental status adult ED patients. This study aims to address this critical knowledge gap and provide evidence to support improved triage and prognostication in emergency care environments.

## Materials and methods

This study was designed as a prospective cohort study and was conducted at Al-Kadhimiya Teaching Hospital, a major tertiary care center located in Baghdad, Iraq. The study period extended from May 2024 to July 2025. The ED of this hospital serves thousands of patients and is equipped to handle most medical emergencies. The target population for this study was individuals who were 18 years or older and presented to the ED with an altered mental status (defined as a GCS score ≤14). All patients had to have been assessed with the FOUR score and GCS score upon admission. Patients who left the hospital against medical advice (LAMA), those with traumatic brain injury (TBI) or any trauma-related cause of altered mental status, and patients with severe dementia or chronic neurodegenerative illnesses that could independently affect neurological outcomes were excluded. Additionally, subjects presenting with active seizures at the time of assessment, those who had received neuromuscular blocking agents or sedatives prior to scoring, and patients with known intellectual disabilities were not included in the study.

Sample size calculation

Sample size calculations were made for the comparative analysis of predictive accuracy of the FOUR score and the GCS score for in-hospital mortality. Pilot data had suggested the following areas under the receiver operating characteristic (AUROC) curves: for the FOUR score, 0.85, and for the GCS score, 0.75. A sample size was derived using MedCalc's ROC comparison tool (MedCalc Software Ltd., Ostend, Belgium) [11] with a Type I error rate (α) of 0.05, 80% power (1-β), and 0.7 correlation between the scores in both the mortality and survival groups. The negative cases were in a ratio of 4:1 to the positive. It was found out from the calculations that there will be at least 48 patients with in-hospital death and 192 surviving patients combined to identify a statistically significant difference between the two AUROC curves. Thus, the total patient sample was 240.

Ethical considerations

This study was reviewed and approved by the Research Ethical Committee, Al-Nahrain College of Medicine, Baghdad, Iraq (approval number: UNCOMIRB20240520, dated May 22, 2024). The study was conducted in accordance with the ethical standards of the Declaration of Helsinki. Informed consent was obtained from all participants (or their legal guardians when applicable) prior to inclusion in the study.

Data collection

Data collection commenced after obtaining ethical approval from the Institutional Review Board (IRB) of Al-Nahrain College of Medicine. This study was also registered prospectively at ClinicalTrials.gov (registration ID: NCT06703619) for purposes of transparency, accountability, and conformity to the international research standards. Prospectively collecting data from eligible patients who showed up at the ED using a structured data collection form. The variables recorded are demographic, such as age and sex, and clinical presentation details, including the chief complaint, diagnosis, and duration of the symptom. Neurological status was assessed through the GCS and FOUR score at admission. In addition to all clinical variables, we recorded the complete timeline of care, including the actual date of admission, death, ICU entry, and discharge.

All data were anonymized and verified for completeness and accuracy before statistical analysis. A three-day training program was conducted for emergency medicine residents to ensure standardized application of the GCS and FOUR scores. Practical sessions were led by two faculty members from the ED. Verbal consent was obtained from the patient or their legal representatives.

Scoring system

The GCS and FOUR score are standardized tools for neurological assessment in determining consciousness (Table 1). GCS, in widespread use for decades, assesses three components: eye opening, verbal response, and motor response, with total scores ranging from 3 to 15. GCS is simple in application and widely accepted, but it has certain limitations in intubated patients and in those who are unable to speak ​​​​​​[12-14]. On the other hand, the FOUR score was designed to resolve some of these limitations and extend a comprehensive neurological assessment. It consists of four domains: eye response, motor response, brainstem reflexes, and respiratory pattern, which are each scored from 0 to 4, with an overall score ranging from 0 to 16 [4,6,15]. The FOUR score evaluates responses from the brainstem and respiratory function, making it especially important for use with critically ill and intubated patients, as it can be utilized where the GCS cannot assess a verbal response.

**Table 1:** Components and Scoring System of the FOUR and GCS Scores GCS: Glasgow Coma Scale; FOUR: Full Outline of UnResponsiveness Source: [4, 12]

FOUR score	Points	GCS score	Points
Eye response		Eye response	
Eyelids open or opened, tracking, or blinking to command	4	Spontaneously	4
Eyelids open but not tracking	3	To verbal command	3
Eyelids closed but open to a loud voice	2	To pain	2
Eyelids closed but open to pain	1	No eye-opening	1
Eyelids remain closed with pain	0	Verbal response	
Motor response		Oriented	5
Thumbs-up, fist, or peace sign	4	Confused	4
Localizing to pain	3	Inappropriate words	3
Flexion response to pain	2	Incomprehensible sounds	2
Extension response to pain	1	No verbal response	1
No response to pain or generalized myoclonus status	0	Motor response	
Brainstem reflexes		Obeys commands	6
Pupil and corneal reflexes present	4	Localizes pain	5
One pupil wide and fixed	3	Withdrawal from pain	4
Pupil OR corneal reflex absent	2	Flexion to pain	3
Pupil AND corneal reflexes absent	1	Extension to pain	2
Absent pupil, corneal, and cough reflexes	0	No motor response	1
Respiration pattern			
Not intubated, regular breathing pattern	4		
Not intubated, Cheyne-Stokes breathing pattern	3		
Not intubated, irregular breathing	2		
Breathes above the ventilatory rate	1		
Breathes at a ventilator rate or apnea	0		

Outcome measures

The primary outcome was in-hospital mortality. Secondary outcomes included the endotracheal intubation and length of hospital stay.

Statistical analysis

Following organization and importation into Microsoft Excel 2019 (Microsoft Corp., Redmond, WA) for preliminary data review and cleaning, statistical analyses were performed on the final dataset using IBM SPSS Statistics software, version 26 (IBM Corp., Armonk, NY). Continuous variables were tested for normality with the Shapiro-Wilk test. Normally distributed variables are reported as mean ± standard deviation (SD), whereas non-normal variables are presented as median with IQR. Categorical variables, such as gender, age group, chief complaints, and duration of altered mental status, are shown as frequencies and percentages. The chi-square test examined the association between the variables and survival outcomes (survival vs. non-survival). To compare GCS and FOUR scores concerning in-hospital mortality, endotracheal intubation, and length of hospital stay, the Mann-Whitney U test was used due to the non-parametric distribution of the data. The predictive accuracy of GCS and FOUR scores was assessed through ROC curves, using the AUROC curve [16]. The AUROCs of each score were compared using the method described by DeLong et al. [16]. Optimal cut-off values were determined, with corresponding sensitivity, specificity, positive predictive value (PPV), and negative predictive value (NPV) reported for each cut-off. The correlation between both scores was assessed using Spearman's rank-order correlation. A p-value of less than 0.05 was considered statistically significant.

## Results

**Figure 1:**
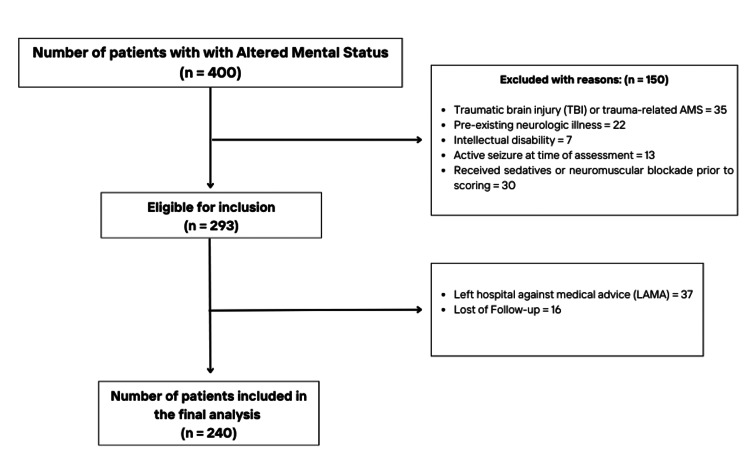
A flow diagram depicting the screening and inclusion of subjects AMS: altered mental status

Of 400 patients screened for eligibility, a total of 240 patients were finally included in the analysis (Figure 1). Females constituted the majority of the study population (55.0%). The median age of participants was 70.0 years (IQR: 21 years). Clinically, decreased level of consciousness (DLOC) was the most common presenting complaint (34.2%). Stroke emerged as the leading ED diagnosis (35.0%) (Table 2).

**Table 2:** Patients’ characteristics DLOC: decreased level of consciousness; SOB: shortness of breath; ED: emergency department; ICH: intracerebral hemorrhage; CKD: chronic kidney disease

Variables	Number	Percentage (%)
Total	240	100%
Gender		
Male	108	45.0%
Female	132	55.0%
Associated symptoms		
DLOC	82	34.2%
SOB	29	12.1%
Aphasia	23	9.6%
Right side weakness	19	7.9%
Abnormal body movement	15	6.3%
Headache	15	6.3%
Left side weakness	11	4.6%
Fever	8	3.3%
Dizziness	8	3.3%
Poor nutrition	7	2.9%
Confusion	7	2.9%
Syncope	6	2.5%
Vomiting and diarrhea	4	1.7%
Abdominal pain	3	1.3%
Anuria	2	0.8%
Melena	1	0.4%
Duration of altered mental status		
0-12 hours	90	37.5%
12-24 hours	65	27.1%
1-3 days	45	18.8%
4-7 days	21	8.8%
More than 7 days	19	7.9%
ED diagnosis		
Stroke	84	35.0%
Infective causes	43	17.9%
ICH/hemorrhages	31	12.9%
Cancer	20	8.3%
Cardiac causes	16	6.7%
Seizure	13	5.4%
Drugs and toxins	7	2.9%
CKD	7	2.9%
Hepatic/uremic encephalopathy	7	2.9%
Hypoglycemia	6	2.5%
Electrolyte disturbance	2	0.8%
Pulmonary diseases	2	0.8%
Gastroenteritis	2	0.8%

Older age (>70 years), longer duration of altered mental status, and lower GCS and FOUR scores were significantly associated with higher mortality (p < 0.05). ICU admission was strongly linked to non-survival, with 88.1% mortality among admitted patients (p < 0.001). Gender showed no significant association with outcome (p = 0.127) (Table 3).

**Table 3:** Sociodemographic and clinical characteristics and their association with clinical outcomes GCS: Glasgow Coma Scale; FOUR: Full Outline of UnResponsiveness

	N (%)	Non-survival outcomes n (%)	Survival outcomes n (%)	Chi-squarevalue	p-value
Total	240 (100%)	95 (39.6%)	145 (60.4%)		
Gender					
Male	108 (45.0%)	37 (34.3%)	71 (65.7%)	2.327	0.127
Female	132 (55.0%)	58 (43.9%)	74 (56.1%)		
Age					
18-40 years	32 (13.3%)	8 (25.0%)	24 (75.0%)	9.233	0.01
40-70 years	109 (45.4%)	37 (33.9%)	72 (66.1%)		
> 70 years	99 (41.3%)	50 (50.5%)	49 (49.5%)		
GCS score					
Below 8	118 (49.2%)	70 (59.3%)	48 (40.7%)	42.115	<0.001
9 to 12	76 (31.7%)	21 (27.6%)	55 (72.4%)		
13 and highest	46 (19.2%)	4 (8.7%)	42 (91.3%)		
FOUR score					
13-16	97 (40.4%)	10 (10.3%)	87 (89.7%)	78.824	<0.001
10-12	58 (24.2%)	24 (41.4%)	34 (58.6%)		
6-9	58 (24.2%)	36 (62.1%)	22 (37.9%)		
<5	27 (11.3%)	25 (92.6%)	2 (7.4%)		
Admission to ICU					
Yes	67 (27.9%)	59 (88.1%)	8 (11.9%)	91.334	<0.001
No	173 (72.1%)	36 (20.8%)	137 (79.2%)		

**Figure 2:**
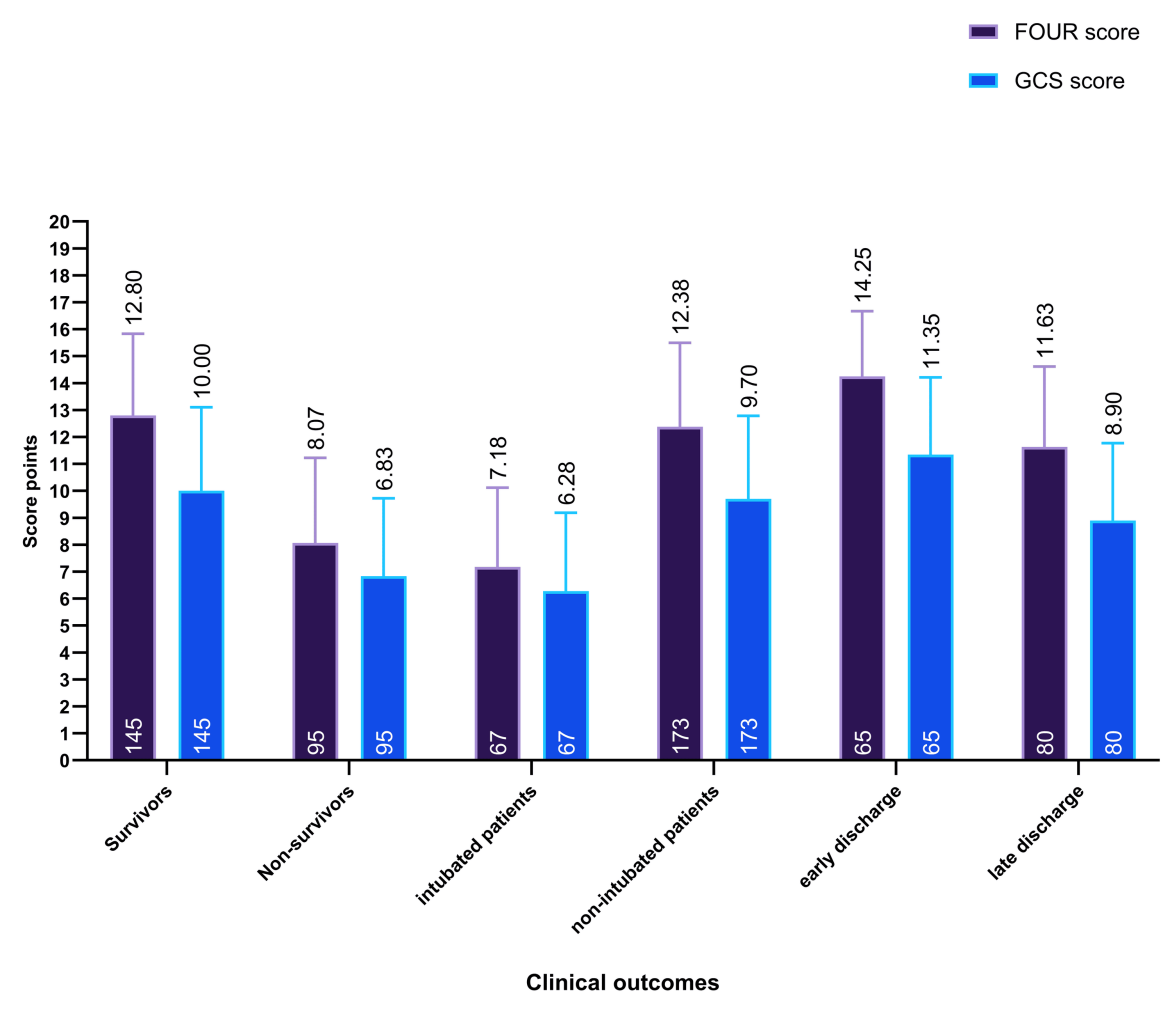
Comparison of FOUR and GCS scores across clinical outcome groups GCS: Glasgow Coma Scale; FOUR: Full Outline of UnResponsiveness

Analysis of clinical-outcome groups showed that both the FOUR score and the GCS were significantly higher in patients with favorable outcomes (Figure 2). Survivors had a mean FOUR score of 12.8 ± 3.04 versus 8.07 ± 3.16 in non-survivors; their mean GCS was 10.0 ± 3.11 compared with 6.83 ± 2.90 in non-survivors. Patients who required endotracheal intubation exhibited the lowest mean scores (FOUR 7.18 ± 2.94; GCS 6.28 ± 2.91), while patients not admitted to the ICU showed higher scores (FOUR 12.38 ± 3.12; GCS 9.70 ± 3.09). By length of stay, those with prolonged hospitalization (>7 days) had lower mean scores (FOUR 11.63 ± 2.99; GCS 8.90 ± 2.87), whereas early-discharge (<3 days) patients had the highest scores (FOUR 14.25 ± 2.42; GCS 11.35 ± 2.87). These differences align consistently across outcomes, supporting the association between higher consciousness scores and better clinical trajectories.

**Figure 3:**
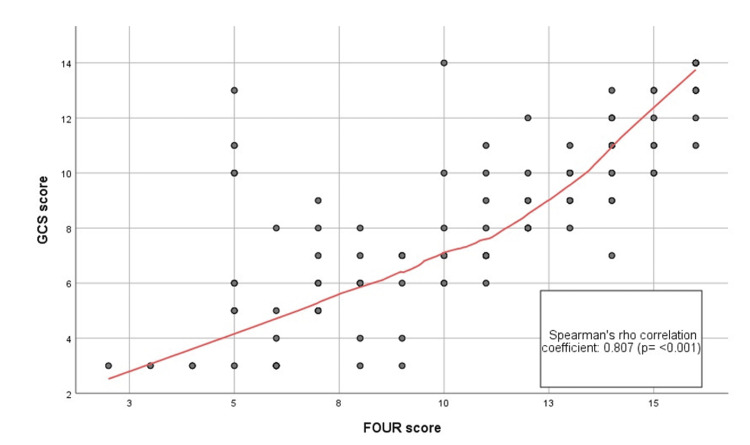
Scatter diagram of Spearman's rho rank correlation between GCS score and FOUR score GCS: Glasgow Coma Scale; FOUR: Full Outline of UnResponsiveness

A Spearman's rank-order correlation was conducted to assess the relationship between the FOUR score and the GCS score. The analysis revealed a strong, positive correlation between the two scores, which was statistically significant (Spearman's ρ = 0.807, p < 0.001). This suggests that higher values on the FOUR score are associated with higher values on the GCS score, and vice versa, as illustrated by the scatter diagram (Figure 3).

**Figure 4:**
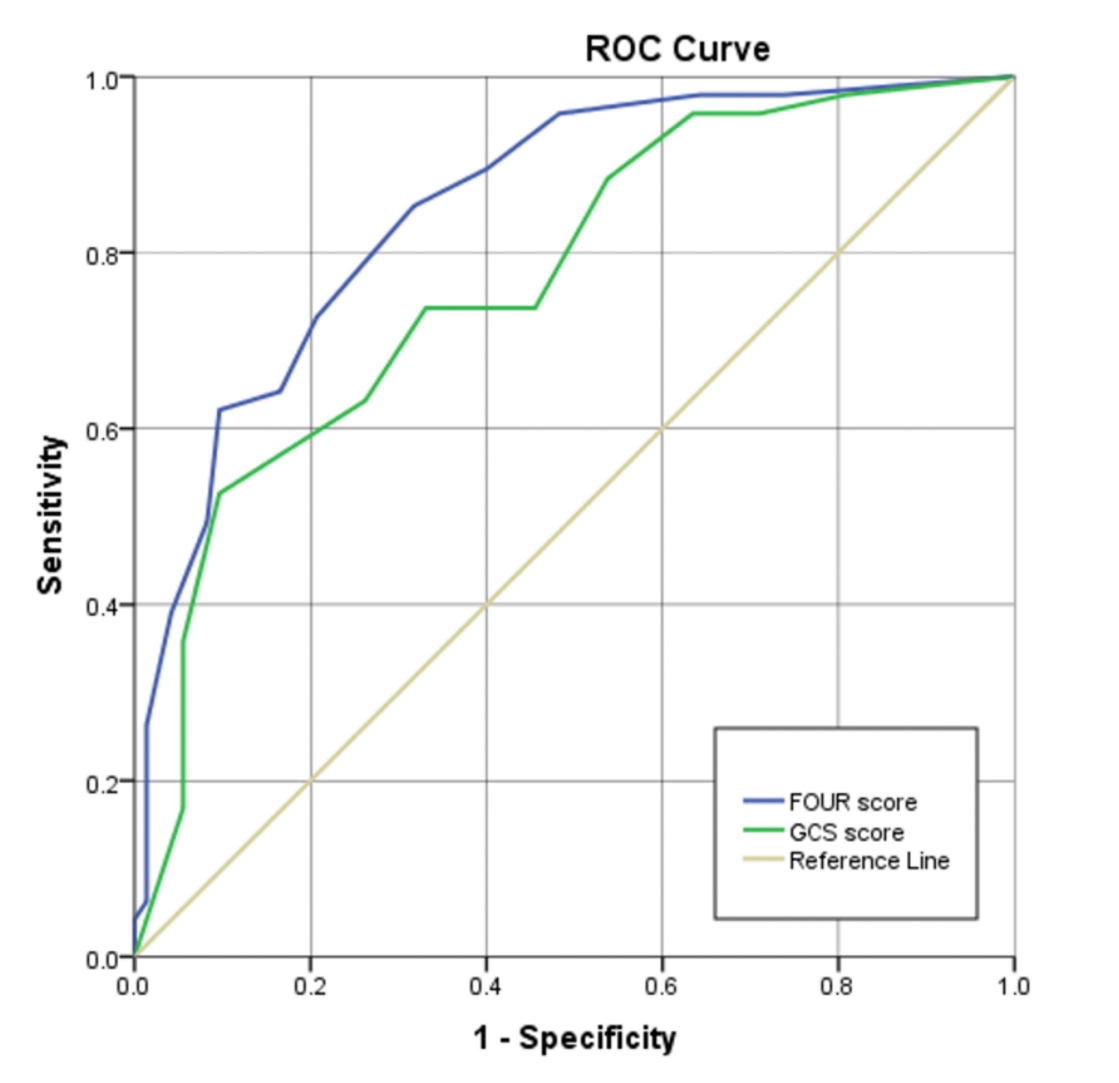
ROC curves of GCS and FOUR score in predicting in-hospital mortality ROC: receiver operating characteristic; GCS: Glasgow Coma Scale, FOUR: Full Outline of UnResponsiveness

Both the FOUR and GCS scores demonstrated significant discriminative ability across all clinical outcomes (all p < 0.001). For in-hospital mortality prediction, the FOUR score (AUROC = 0.851, 95% CI: 0.803-0.900) outperformed the GCS (AUROC = 0.771, 95% CI: 0.710-0.831). At optimal cut-offs (FOUR: 10; GCS: 8), the FOUR score achieved higher specificity (79.3% vs. 66.9%) and a superior negative predictive value (81.6% vs. 79.5%), though with slightly lower sensitivity (72.6% vs. 73.7%). The positive predictive value was also higher for the FOUR score (69.7% vs. 59.3%) (Figure 4 and Table 4).

**Table 4:** Diagnostic values of GCS and FOUR score for the prediction of in-hospital mortality GCS score: Glasgow Coma Scale; FOUR: Full Outline of UnResponsiveness; AUC: area under the receiver operating characteristic (ROC) curve

	GCS score	FOUR score
AUC	0.771	0.851
Confidence interval	(0.710 - 0.831)	(0.803 – 0.900)
p-value	<0.001	<0.001
Cutoff	8	10
Sensitivity	73.70%	72.60%
Specificity	66.90%	79.30%
Positive predictive value	59.3%	69.7%
Negative predictive value	79.5%	81.6%

**Figure 5:**
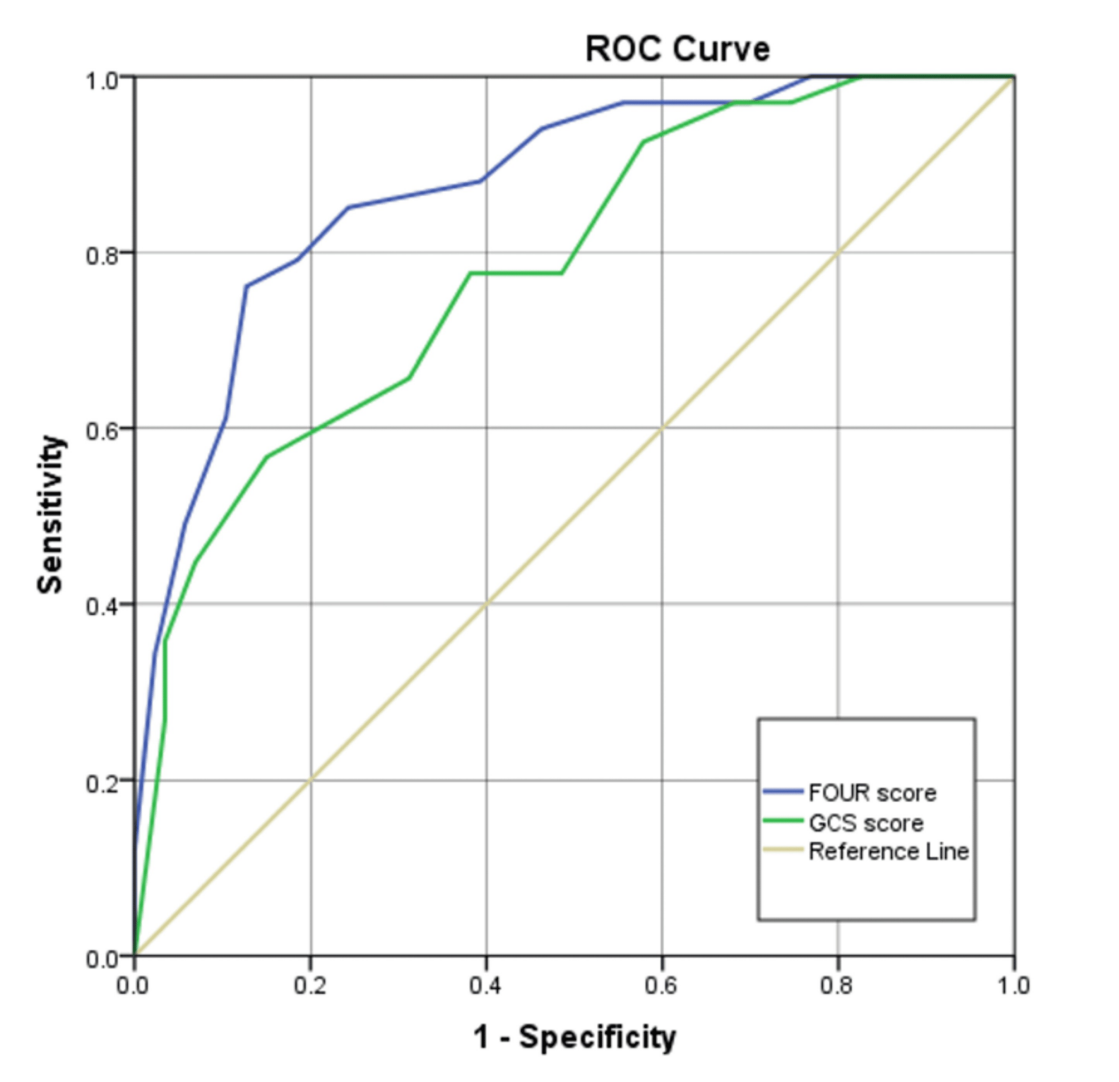
ROC curves of GCS and FOUR score in predicting endotracheal intubation ROC: receiver operating characteristic; GCS: Glasgow Coma Scale, FOUR: Full Outline of UnResponsiveness

For prediction of endotracheal intubation, the FOUR score again showed superior discrimination (AUROC = 0.875, 95% CI: 0.827-0.923) compared to the GCS (AUROC = 0.781, 95% CI: 0.717-0.845), both p <0.001. At optimal cut-offs (FOUR: 10; GCS: 8), the FOUR score demonstrated higher sensitivity (85.1% vs. 77.6%) and specificity (75.7% vs. 61.8%), as well as superior predictive values (PPV: 57.6% vs. 44.1%; NPV: 92.9% vs. 87.7%) (Figure 5 and Table 5).

**Table 5:** Diagnostic values of GCS and FOUR score for the prediction of endotracheal intubation GCS score: Glasgow Coma Scale; FOUR: Full Outline of UnResponsiveness; AUC: area under the receiver operating characteristic (ROC) curve

	GCS score	FOUR score
AUC	0.781	0.875
Confidence interval	(0.717 - 0.845)	(0.827 - 0.923)
p-value	<0.001	<0.001
Cutoff	8	10
Sensitivity	77.60%	85.10%
Specificity	61.80%	75.70%
Positive predictive value	44.1%	57.6%
Negative predictive value	87.7%	92.9%

**Figure 6:**
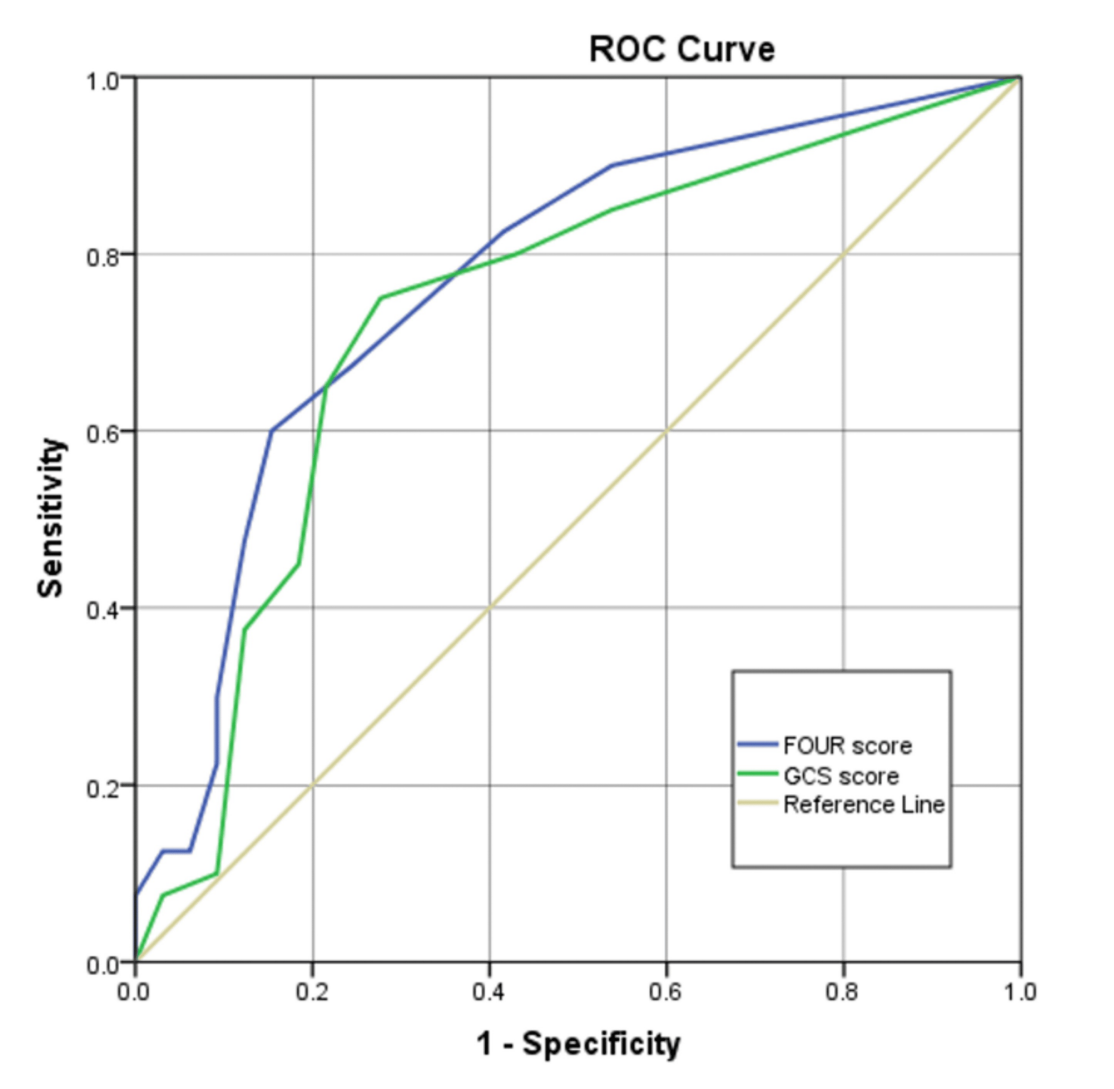
ROC curves of GCS and FOUR score in predicting the length of hospital stay ROC: Receiver operating characteristic; GCS: Glasgow Coma Scale, FOUR: Full Outline of UnResponsiveness

For the prediction of the length of hospital stay, both the GCS and FOUR scores demonstrated significant discriminative ability (p < 0.001) to predict prolonged length of hospitalization. The FOUR score (AUROC = 0.771, 95% CI: 0.692-0.850) outperformed the GCS (AUROC = 0.734, 95% CI: 0.649-0.820). At optimal cut-offs (FOUR: 14; GCS: 10), the FOUR score showed higher sensitivity (82.5% vs. 75.0%) and a better negative predictive value (73.1% vs. 70.1%), while the GCS achieved superior specificity (72.3% vs. 58.5%) and positive predictive value (76.9% vs. 71.0%) (Figure 6 and Table 6).

**Table 6:** Diagnostic values of GCS and FOUR score for prediction of length of hospital stay GCS score: Glasgow Coma Scale; FOUR: Full Outline of UnResponsiveness; AUC: area under the receiver operating characteristic (ROC) curve

	GCS score	FOUR score
AUC	0.734	0.771
Confidence interval	0.649 - 0.820	0.692 - 0.850
p-value	<0.001	<0.001
Cutoff	10	14
Sensitivity	75%	82.5%
Specificity	72.3%	58.5%
Positive predictive value	76.9%	71.0%
Negative predictive value	70.1%	73.1%

## Discussion

To the authors’ knowledge, this study represents the first global investigation to validate the FOUR score and compare it with the GCS in predicting endotracheal intubation and length of hospital stay, as well as the first study from the Middle East and North Africa (MENA) region to evaluate its utility in predicting in-hospital mortality among adult patients presenting with non-traumatic altered mental status in the ED. The findings demonstrated that the FOUR score provided superior predictive ability for in-hospital mortality, the need for endotracheal intubation, and length of hospital stay compared with the GCS.

The present study found that the FOUR score outperformed the GCS in predicting in-hospital mortality, with AUROC values of 0.851 and 0.771, respectively. This finding is consistent with several previous studies that have demonstrated the superior predictive validity of the FOUR score over the GCS [6,17,18]. For instance, Wijdicks et al. reported AUROC values of 0.742 for the FOUR score versus 0.715 for the GCS in critically ill ICU patients [17]. Similarly, Eken et al., in a cohort of 185 patients, found AUROCs of 0.788 for the FOUR score compared to 0.735 for the GCS [18]. Similar findings were reported in studies conducted among populations with TBI by Chattopadhyay et al. [19], Anand et al. [20], and Okasha et al. [10].

However, not all studies have observed this advantage. In a prospective cohort study of 359 patients with non-traumatic altered mental status in the ED, Abdallah et al. reported no significant difference between the two scales, with AUROCs of 0.68 for the FOUR score and 0.67 for the GCS in predicting 30-day mortality [21]. Similarly, Pandey et al. and Stead et al. found comparable performance between the two scores [7, 22]. These discrepancies may reflect methodological and contextual differences. Unlike our study, which focused on in-hospital mortality, Abdallah et al. assessed 30-day mortality as the primary outcome. Furthermore, variations in patient demographics, baseline neurological status, and clinical setting could have influenced the relative predictive accuracy of each scoring system.

Both the GCS and the FOUR score demonstrated high accuracy in predicting the need for endotracheal intubation, with the FOUR score showing superior discriminatory ability (AUROC = 0.875) compared with the GCS (AUROC = 0.781). The limited number of studies evaluating the validity of the FOUR score in predicting intubation renders the current evidence inconclusive. However, a study by Okasha et al., conducted among 60 TBI patients admitted to the ICU, reported no significant difference between the two scoring systems in predicting intubation (AUC 0.961 vs. 0.982, p = 0.06) [10]. These findings are in contrast to the present study, which demonstrated the superiority of the FOUR score. The discrepancy may be attributed to differences in study populations and settings, as Okasha et al. focused on ICU patients with TBI, whereas our study involved non-traumatic patients presenting to the ED. In addition, the sample size in Okasha et al.’s study was relatively small compared with our larger cohort, which may also account for the variation in results.

In endotracheal intubation, structured neurological tools such as the FOUR score are particularly valuable in settings with limited ICU resources. Unlike the GCS, the FOUR score incorporates respiratory patterns and brainstem reflexes, improving the accuracy of triage decisions and resource allocation. Its standardized use also facilitates continuity of care, daily re-evaluation, and early detection of neurological deterioration, while supporting outcome prediction and brain death diagnosis.

Length of stay is an important measure of healthcare utilization and a key determinant of hospitalization costs. Although numerous studies have evaluated various scoring systems for predicting mortality, relatively few have investigated their ability to predict length of stay, particularly in the ED setting. In the present study, both the FOUR score and GCS demonstrated statistically significant predictive value, with the FOUR score showing higher accuracy (AUROC = 0.771 vs. 0.734). Consistent with our findings, Anand et al., in a cohort of 107 TBI patients, reported that the FOUR score was a better predictor of hospital stay compared with the GCS [20]. Conversely, Okasha et al., in a study of TBI patients admitted to the ICU, found no significant difference between the two scores in predicting ICU length of stay [10]. This discrepancy may be attributed to differences in study populations (ICU TBI patients vs. non-traumatic altered mental status patients in the ED), sample sizes (60 vs. 240 patients), and outcome definitions (ICU stay vs. total hospital stay).

The higher accuracy of the FOUR score compared to the GCS in predicting endotracheal intubation, hospital length of stay, and in-hospital mortality may be explained by several factors. First, the inclusion of brainstem reflexes and respiratory pattern assessment in the FOUR score provides additional clinical information beyond motor and verbal responses, which are often limited in intubated or deeply obtunded patients [6,10,23]. Second, the FOUR score avoids the verbal response component of the GCS, reducing subjectivity and improving applicability in patients with impaired speech or on mechanical ventilation [4, 6]. Third, by offering a broader scoring spectrum at the lower end of consciousness assessment, the FOUR score allows for better discrimination among patients with the lowest GCS scores, thereby refining prognostication [4]. Chen et al. in a study of 101 neurosurgical patients with GCS scores reported a higher AUC for FOUR scores than GCS in predicting mortality (0.768 vs. 0.699) and concluded that the difference in predictive power between FOUR scores and GCS is larger in patients with low scores [24].

Limitations

Several limitations of this study should be acknowledged. First, the study was conducted at a single center, which may limit the generalizability of the findings. However, research on altered mental status in ED settings in low- and middle-income countries (LMICs) is challenging, and this study represents an important contribution to addressing this gap. Second, we did not evaluate long-term outcomes of our study population due to difficulties in follow-up in this context. Third, variability in pre-hospital care and delays in presentation may have influenced initial neurological status and score performance. Finally, we did not assess the inter-rater reliability of the two scores in our setting, but numerous previous studies used by providers with little training have shown excellent inter-rater reliability [25, 26].

## Conclusions

The FOUR score outperformed the GCS in predicting in-hospital mortality, endotracheal intubation, and hospital length of stay in non-traumatic altered mental status. These findings advocate for the incorporation of the FOUR score into emergency triage protocols to enhance resource allocation and clinical decision-making in resource-constrained environments. However, further multicenter and larger-scale studies are warranted to validate these results and explore the applicability of the FOUR score across diverse patient populations and healthcare settings.
